# Clinical Characteristics and Outcomes of a High-grade Endometrial Cancer Cohort Treated at Instituto Nacional de Câncer, Brazil

**DOI:** 10.1055/s-0043-1772177

**Published:** 2023-08-18

**Authors:** Eduardo Paulino, Andreia Cristina de Melo

**Affiliations:** 1 Instituto Nacional de Câncer, Rio de Janeiro, RJ, Brazil; 2 Oncologia D'or, Rio de Janeiro, RJ, Brazil; 3 Grupo Oncoclínicas, Rio de Janeiro, RJ, Brazil

**Keywords:** endometrial cancer, endometrial cancer therapy, endometrial cancer pathology, demographic analysis, câncer de endométrio, terapia para câncer de endométrio, patologia câncer de endométrio, análise demográfica

## Abstract

**Objective** To analyze the outcomes of a cohort of patients with high-risk histologies of endometrial cancer (EC) treated at Instituto Nacional de Câncer (National Cancer Institute, INCA, in Portuguese), in Brazil.

**Materials and Methods** We reviewed the medical records of patients with high-risk histologies of EC in any stage registered at INCA between 2010 and 2016 to perform a clinical and demographic descriptive analysis and to evaluate the outcomes in terms of recurrence and survival.

**Results** From 2010 to 2016, 2,145 EC patients were registered and treated at INCA, and 466 had high-grade histologies that met the inclusion criteria. The mean age of the patients was 65 years, 44.6% were Caucasian, and 90% had a performance status of 0 or 1. The most common histology was high-grade endometrioid (31.1%), followed by serous carcinoma (25.3%), mixed (20.0%), carcinosarcoma (13.5%), and clear cell carcinoma (9.4%). Considering the 2018 Fédération Internationale de Gynécologie et d'Obstétrique (International Federation of Gynecology and Obstetrics, FIGO, in French) staging system, 44.8%, 12.4%, 29.8%, and 12.9% of the patient were in stages I, II, III or IV respectively. Age (> 60 years), more than 50% of myoinvasion, higher stage, poor performance status, serous and carcinosarcoma histologies, and adjuvant treatment were independent factors associated with recurrence-free survival (RFS) and overall survival (OS) in the multivariate analysis.

**Conclusion** The current findings reinforced the international data showing poor outcomes of these tumors, especially for serous and carcinosarcomas and tumors with advanced stages, with shorter survival and high recurrence rates in distant sites, independently of the FIGO stage. Adjuvant therapy was associated with better survival.

## Resumo

**Objetivo** Analisar os desfechos de uma coorte de pacientes com câncer de endométrio (CE) e histologias de alto risco atendida no Instituto Nacional do Câncer (INCA) entre 2010 e 2016.

**Materiais e Métodos** Foram revisados prontuários de pacientes com histologias de alto risco de CE em qualquer estágio cadastradas no INCA entre 2010 e 2016 para realizar uma análise descritiva clínica e demográfica e avaliar os resultados em termos de recorrência e sobrevida.

**Resultados** De 2010 a 2016, 2.145 pacientes com CE foram cadastradas e atendidas no INCA, e 466 tinham histologias de alto grau e atendiam aos critérios de inclusão. A média de idade das pacientes foi de 65 anos, 44,6% eram brancas, e 90% tinham *performance status* de 0 ou 1. A histologia mais comum foi endometrioide de alto grau (31,1%), seguida de carcinoma seroso (25,3%), misto (20,0%), carcinossarcoma (13,5%) e carcinoma de células claras (9,4%). Considerando o estadiamento da Fédération Internationale de Gynécologie et d'Obstétrique (Federação Internacional de Ginecologia e Obstetrícia, FIGO, em francês) de 2018, 44,8%, 12,4%, 29,8% e 12,9% apresentaram estágios I, II, III ou IV, respectivamente. Idade (> 60 anos), mais de 50% de mioinvasão, estágio avançado, *performance status* ruim, histologias serosas e carcinossarcoma, e tratamento adjuvante foram fatores independentes associados à sobrevida livre de recorrência e sobrevida global na análise multivariada.

**Conclusão** Os achados atuais reforçam os dados internacionais que demonstram o prognóstico ruim desses tumores, principalmente para as histologias serosas e carcinossarcomas e para estágios avançados, com menor sobrevida e altas taxas de recorrência à distância, independentemente do estágio da FIGO. A terapia adjuvante foi associada a melhor sobrevida.

## Introduction

Endometrial cancer (EC) is the most common gynecological cancer in developed countries and the third most common gynecological cancer in Brazil. Approximately 6,500 new cases were expected in Brazilian women per year from 2020 to 2022 and it caused 1,823 deaths in 2020. 1 The worldwide incidence is expected to increase in upcoming years, since obesity and aging, which are important risk factors to develop EC, are increasing. 2

In 1983, Bohkman 3 performed a prospective study dividing EC into two major subgroups, in a classification that was used for decades. The first subgroup, named type I, was characterized by lower-grade histology, less aggressive behavior, metabolic syndrome, and a better response to progestins. The second, type II, with more aggressive behavior, is more commonly diagnosed in the advanced stage and is less responsive to progestins. At that time, Bohkman 3 did not know the different histologies described years later with the classification by the World Health Organization (WHO): endometrioid, serous, clear-cell, and undifferentiated carcinomas. 4 It is well known that serous carcinoma, clear-cell carcinoma (CCC), and carcinosarcoma (CCS) have a poorer prognosis compared with endometrioid tumors, and they are classified as type-II tumors. 5 6 However, high-grade endometrioid tumors are more heterogeneous and sometimes can show intermediate or similar prognosis to that of serous carcinomas and CCCs. 5 6 7 8 In recent years, this dualist vision of EC has been replaced by a more accurate molecular profile. In this system, developed by The Cancer Genome Atlas (TCGA) using next-generation sequencing, patients with endometrioid and serous carcinomas were classified in one of the four molecular subgroups: those with mutations in the *POLE* gene (called “POLE ultramutated”, the subgroup with good prognosis); those with *p53* gene mutation (called “copy number high”, the subgroup with poor prognosis), those with mutations in the *MLH1* , *MSH2* , *MSH6* or *PMS2* genes (called “microssatelite instability”), and those without a characteristic gene mutation (called “copy number low”), both with an intermediate prognosis. 9

There is a paucity of information about the prognosis and outcomes of EC patients in the Brazilian population. Thus, the present study aimed to analyze demographic and prognostic characteristics of a high-grade EC cohort treated in a tertiary cancer center in Brazil.

## Materials and Methods

The present is a retrospective study conducted at Instituto Nacional de Câncer (National Cancer Institute, INCA, in Portuguese). The medical records of EC patients registered at INCA between 2010 and 2016 were reviewed. The inclusion criteria were patients over 18 years of age with high-grade endometrioid carcinomas, serous carcinomas, CCCs, CCSs or undifferentiated adenocarcinomas, regardless of the stage (I-IV) according to the 2018 Fédération Internationale de Gynécologie et d'Obstétrique (International Federation of Gynecology and Obstetrics, FIGO, in French) staging system, who underwent the whole treatment at INCA. The exclusion criteria were patients with other synchronous or metachronous primary tumors, non-epithelial histologies, and low-grade histologies. Sociodemographic (age and race) and clinical variables (histology type, stage, treatment received, recurrence, type of recurrence, death) were collected.

The primary objective of the present study was to perform a clinical and demographic descriptive analysis and to evaluate the outcomes in terms of recurrence and survival of this cohort. Pelvic recurrences included vaginal and local recurrences (including to the pelvic lymph nodes and local spread to the rectum and bladder); recurrences outside the pelvis consisting of peritoneal carcinomatosis or omental metastasis were classified as abdominal recurrences; distant hematogenous recurrences include lung, liver, bone, and brain metastases, as well as non-pelvic or para-aortic lymph node involvement. Simultaneous pelvic and abdominal recurrences were classified as abdominal recurrence; simultaneous abdominal and distant recurrences were considered distant recurrence; and simultaneous locoregional and distant recurrences were also considered distant recurrence. Recurrence-free survival (RFS) was defined as the time from diagnosis to the date of recurrence confirmed by imaging or clinically (local or distant), or death, with censoring of patients alive without recurrence. Overall survival (OS) was defined as the time from diagnosis to death, regardless of the cause, with censoring of patients alive on the date of the last follow-up.

Statistical analyses were performed using the R (R Foundation for Statistical Computing, Vienna, Austria) software. Patient and tumor characteristics were compared with the *t* -test for continuous variables and with the Chi-squared (χ ^2^ ) or Fisher exact tests for categorical variables. Rates of distant and locoregional recurrences, and RFS and OS were analyzed by the Kaplan-Meier method and log-rank test from the date of diagnosis, with censoring of patients who were alive and recurrence-free on the date of the last follow-up. For the RFS analysis, all recurrences (locoregional and distant) were considered an event; for OS, all deaths regardless of the cause were considered an event. All statistical tests were two-sided and values of *p* < 0.05 were considered statistically significant.

The present study was approved by the institutional ethical committee (under number 26543019.5.0000.5274) and is in accordance with the Good Clinical Practice.

## Results

From 2010 to 2016, 2,145 EC patients were registered and treated at INCA. Of these, 466 had high-grade histologies and met all the inclusion criteria. Their mean age was 65 years (standard deviation [SD]: ± 11.5), 44.6% were Caucasian, and 90% had a performance status (PS) of 0 or 1. The most common histology was high-grade endometrioid carcinoma (145; 31.1%) followed by serous carcinoma (118; 25.3%), mixed carcinoma (93; 20.0%), CCS (63, 13.5%), and CCC (44; 9.4%). Regarding stage, 44.8%, 12.4%, 29.8% and 12.9% were in 2018 FIGO stages I, II, III and IV respectively, and 48.8% presented more than 50% of myoinvasion.

The surgery route was an open laparotomy for 90.3% of the patients. Most underwent total hysterectomy plus salpingooforectomy and lymphadenectomy (33.8%) or in addition to omentectomy (34.8%); 31.4% were not submitted to lymph node assessment (they underwent only total hysterectomy with or without salpingooforectomy). Minimally-invasive surgery was barely used (laparoscopy: 5.7%; and robotic: 1.7%). Adjuvant treatment was administered to 77.6% of the cohort. Chemotherapy (mostly carboplatin plus paclitaxel) with or without any radiation therapy (external or brachytherapy) was prescribed to 50% of the patients; exclusive external beam radiotherapy and/or brachytherapy were performed in 27.1%. The mean number of cycles of systemic therapy was of 5.7, and the mean doses of external beam radiotherapy and brachytherapy were of 4,800 cGy and 2,675.4 cGy respectively. Table 1 summarizes the main characteristics of the patients.

**Table 1:** Clinical characteristics of the study cohort

Variables		n (%); N =466
Age	Mean (± standard deviation)	65.9 (11.5) years
Age group	< 60	118 (25.3) years
	≥ 60	348 (74.7) years
Race	White	208 (44.6)
	Black	89 (19.1)
	Non-white/black	169 (36.3)
Histological subtype	AEG3 USC CCC CCS Mixed NOS	145 (31.1) 118 (25.3) 44 (9.4) 63 (13.5) 93 (20.0) 3 (0.6)
Type of surgery	TAH	14 (3.0)
	TAH + BSO	132 (28.4)
	TAH + BSO + LFN (PLNs or PALNs )	157 (33.8)
	TAH + BSO + LFN + omentectomy	162 (34.8)
Route of surgery	Open	420 (90.3)
	Laparoscopic	25 (5.4)
	Transvaginal	12 (2.6)
	Robotic	8 (1.7)
ECOG-PS	0	116 (25.3)
	1	297 (64.8)
	2	33 (7.2)
	3	12 (2.6)
2018 FIGO	IA	148 (31.8)
	IB	61 (13.1)
	II	58 (12.4)
	IIIA	35 (7.5)
	IIIB	6 (1.3)
	IIIC1	63 (13.5)
	IIIC2	35 (7.5)
	IVA	2 (0.4)
	IVB	58 (12.4)
2018 FIGO	I	209 (44.8)
	II	58 (12.4)
	III	139 (29.8)
	IV	60 (12.9)
Myoinvasion	No invasion	24 (5.2)
	Less than 50%	214 (46.0)
	More than 50%	227 (48.8)
Chemotherapy	No	231 (49.6)
	Yes	235 (50.4)
Type of chemotherapy	Adjuvant	225 (95.7)
	Neoadjuvant	10 (4.3)
Cycles of chemotherapy	Mean (± standard deviation )	5.7 (1.1)
Adjuvant radiotherapy	No	313 (67.3)
	Yes	152 (32.7)
Radiotherapy dose (cGY)	Mean (± standard deviation )	4,800 (2,600)
Adjuvant brachytherapy	No	326 (70.1)
	Yes	139 (29.9)
Brachytherapy dose (cGY)	Mean (± standard deviation )	2,675.4 (350.6)
Any type of treatment ^a^	No	104 (22.4)
	Yes	361 (77.6)
Type of treatment	No treatment	104 (22.4)
	Chemotherapy with or without EBR/BT	235 (50.5)
	EBR and/or BT	126 (27.1)
Disease recurrence	No	261 (56.1)
	Yes	204 (43.9)
Site of recurrence	Pelvis	60 (29.4)
	Abdomen	54 (26.5)
	Distant	90 (44.1)
Death	No	200 (42.9)
	Yes	266 (57.1)

Abbreviations: BSO bilateral salpingooforectomy; BT, brachytherapy; CCC, clear-cell carcinoma; CCS; carcinosarcoma; EBR, external beam radiotherapy; ECOG-PS, Eastern Cooperative Oncology Group's Performance Status scale; FIGO, Fédération Internationale de Gynécologie et d'Obstétrique (International Federation of Gynecology and Obstetrics, in French) staging system; G3EA, grade-3 endometrioid adenocarcinoma; LFN, lymphadenectomy; x Mixed, mixed epithelial tumors; NOS, not otherwise specified; PALNs, paraaortic lymph nodes; PLNs, pelvic lymph nodes; TAH, total abdominal hysterectomy; USC, uterine serous carcinoma.

Note: ^a^ Includes chemotherapy and/or radiotherapy and/or brachytherapy.

The median follow-up was of 74.9 months. As expected, recurrence was quite common in this high-risk cohort, with a rate of 43.8%. For the whole population, the recurrence pattern was most common in distant sites (44.1%), followed by abdominal (26.5%) and pelvic (29.4%). Focusing on the recurrence pattern by stage or histology, these findings were not different, with most recurrences occurring in distant sites ( Tables 1 and 2 ).

**Table 2:** Patterns of recurrence

		Pelvic – n (%)	Abdominal – n (%)	Distant – n (%)	Total – n (%)	*p*
2018 FIGO	IA	11 (37.9)	5 (17.2)	13 (44.8)	29 (100)	0.537
	IB	3 (25.0)	3 (25.0)	6 (50.0)	12 (100)	
	II	11 (42.3)	2 (7.7)	13 (50.0)	26 (100)	
	IIIA	6 (31.6)	5 (26.3)	8 (42.1)	19 (100)	
	IIIB	3 (60.0)	1 (20.0)	1 (20.0)	5 (100)	
	IIIC1	10 (24.4)	14 (34.1)	17 (41.5)	41 (100)	
	IIIC2	5 (20.0)	8 (32.0)	12 (48.0)	25 (100)	
	IVA	0 (0.0)	0 (0.0)	1 (100.0)	1 (100)	
	IVB	11 (23.9)	16 (34.8)	19 (41.3)	46 (100)	
2018 FIGO	I	14 (34.1)	8 (19.5)	19 (46.3)	41 (100)	0.179
	II	11 (42.3)	2 (7.7)	13 (50.0)	26 (100)	
	III	24 (26.7)	28 (31.1)	38 (42.2)	90 (100)	
	IV	11 (23.4)	16 (34.0)	20 (42.6)	47 (100)	
Histological subtype	G3EA	9 (21.4)	12 (28.6)	21 (50.0)	42 (100)	0.551
	USC	17 (25.0)	18 (26.5)	33 (48.5)	68 (100)	
	CCC	4 (22.2)	5 (27.8)	9 (50.0)	18 (100)	
	CCS	15 (36.6)	11 (26.8)	15 (36.6)	41 (100)	
	Mixed	15 (42.9)	8 (22.9)	12 (34.3)	35 (100)	
Myoinvasion	No invasion	5 (50.0)	3 (30.0)	2 (20.0)	10 (100)	0.343
	Less than 50%	24 (33.3)	16 (22.2)	32 (44.4)	72 (100)	
	More than 50%	31 (25.6)	34 (28.1)	56 (46.3)	121 (100)	
	(Missing)	0 (0.0)	1 (100.0)	0 (0.0)	1 (100)	
Myoinvasion	No or less than 50%	29 (35.4)	19 (23.2)	34 (41.5)	82 (100)	0.320
	More than 50%	31 (25.6)	34 (28.1)	56 (46.3)	121 (100)	
	(Missing)	0 (0.0)	1 (100.0)	0 (0.0)	1 (100)	

Abbreviations: CCC, clear-cell carcinoma; CCS; carcinosarcoma; FIGO, Fédération Internationale de Gynécologie et d'Obstétrique (International Federation of Gynecology and Obstetrics, in French) staging system; G3EA, grade-3 endometrioid adenocarcinoma; Mixed, mixed epithelial tumors; USC, uterine serous carcinoma.

Notes: Pelvic recurrences included vaginal and pelvic recurrences (including pelvic lymph nodes and local spread to the rectum and bladder); recurrences outside the pelvis consisting of peritoneal carcinomatosis or omental metastasis were classified as abdominal recurrences; distant recurrences include lung, liver, bone, and brain metastases, as well as non-pelvic or paraaortic lymph node involvement. Simultaneous locoregional and abdominal recurrences were classified as abdominal recurrence; simultaneous abdominal and distant recurrences were considered as distant recurrence, and simultaneous locoregional and distant recurrences were considered as distant recurrence.

The mean RFS and OS were of 36.8 and 52.8 months respectively. As expected, poor PS was associated with worse outcomes (OS of 131, 90, 64, and 48 months for PS 0,1, 2 and 3 respectively) as well as higher stages (for patients in stages I, II, III, and IV the RFS and OS were of 118 and 122; 37.5 and 48.5; 20.4 and 29.9; and 9.4 and 18 months respectively). Regarding histology, CCS and serous carcinoma had the worse prognosis, followed by CCC, mixed and high-grade endometrioid carcinomas. Age > 60 years, more than 50% of myoinvasion, higher stage, poor PS, serous and CCS histologies, and adjuvant treatment were independent factors associated with RFS and OS in the univariate and multivariate analyses ( Tables 3 and 4 ).

**Table 3:** Univariate and multivariate analyses for overall survival (OS)

Variable – OS		All – n (%)	HR (95%CI; *p* -value) –univariate analysis	HR (95%CI; *p* -value) –multivariate analysis
Age at diagnosis (in years)	Mean (± SD)	65.9 (11.5)	1.04 (1.02–1.05; *p* < 0.001)	−
Age group (in years)	< 60	118 (25.3)	−	−
	≥ 60	348 (74.7)	2.37 (1.71–3.29; *p* < 0.001)	2.05 (1.45–2.89; *p* < 0.001)
Race	White	208 (44.6)	−	−
	Black	89 (19.1)	1.37 (1.00–1.89; *p* = 0.053)	−
	Non-white/black	169 (36.3)	1.23 (0.94–1.61; *p* = 0.139)	−
Race	White	208 (44.6)	−	−
	Non-white	258 (55.4)	1.28 (1.00–1.63; *p* = 0.050)	−
2018 FIGO	I	209 (44.8)	−	−
	II	58 (12.4)	2.26 (1.52–3.36; *p* < 0.001)	2.85 (1.88–4.31; *p* < 0.001)
	III	139 (29.8)	3.08 (2.27–4.16; *p* < 0.001)	4.81 (3.21–7.19; *p* < 0.001)
	IV	60 (12.9)	5.37 (3.75–7.70; *p* < 0.001)	8.03 (5.07–12.73; *p* < 0.001)
ECOG-PS	0	116 (25.3)	−	−
	1	297 (64.8)	3.26 (2.26–4.70; *p* < 0.001)	2.28 (1.55–3.35; *p* < 0.001)
	2	33 (7.2)	5.40 (3.25–8.98; *p* < 0.001)	3.20 (1.87–5.46; *p* < 0.001)
	3	12 (2.6)	8.63 (4.35–17.10; *p* < 0.001)	4.03 (1.90–8.55; *p* < 0.001)
Myoinvasion	No invasion	24 (5.2)	−	−
	Less than 50%	214 (46.0)	1.40 (0.71–2.77; *p* = 0.338)	−
	More than 50%	227 (48.8)	2.76 (1.40–5.41; *p* = 0.003)	−
Myoinvasion	No or less than 50%	238 (51.2)	−	−
	More than 50%	227 (48.8)	2.04 (1.59–2.60; *p* < 0.001)	1.55 (1.18–2.04; *p* = 0.002)
Histological subtype	G3EA	145 (31.1)	−	−
	USC	118 (25.3)	1.90 (1.37–2.64; *p* < 0.001)	1.56 (1.10–2.22; *p* = 0.013)
	CCC	44 (9.4)	1.47 (0.94–2.30; *p* = 0.095)	1.18 (0.73–1.88; *p* = 0.500)
	CCS	63 (13.5)	2.97 (2.04–4.32; *p* < 0.001)	2.35 (1.56–3.52; *p* < 0.001)
	Mixed	93 (20.0)	1.06 (0.72–1.56; *p* = 0.769)	0.92 (0.61–1.38; *p* = 0.674)
	NOS	3 (0.6)	0.00 (0.00–Inf; *p* = 0.991)	0.00 (0.00–Inf; *p* = 0.992)
Treatment	No treatment	104 (22.4)	−	−
	Chemotherapy with or without EBR/BT	235 (50.5)	0.75 (0.56–1.00; *p* = 0.052)	0.30 (0.20–0.44; *p* < 0.001)
	EBR and/or BT	126 (27.1)	0.46 (0.32–0.66; *p* < 0.001)	0.56 (0.38–0.83; *p* = 0.004)

Abbreviations: 95%CI, 95% confidence interval; BT, brachytherapy; CCC, clear-cell carcinoma; CCS; carcinosarcoma; EBR, external beam radiotherapy; ECOG-PS, Eastern Cooperative Oncology Group's Performance Status scale; FIGO, Fédération Internationale de Gynécologie et d'Obstétrique (International Federation of Gynecology and Obstetrics, in French) staging system; G3EA, grade-3 endometrioid adenocarcinoma; HR, hazard ratio; Mixed, mixed epithelial tumors; NOS, not otherwise specified; SD, standard deviation; USC, uterine serous carcinoma.

**Table 4:** Univariate and multivariate analysis for recurrence-free survival (RFS)

Variable – RFS		All – n (%)	HR (95%CI; *p* -value) –univariate analysis	HR (95%CI; *p* -value) –univariate analysis
Age at diagnosis (in years)	Mean (± SD)	65.9 (11.5)	1.03 (1.02–1.04; *p* < 0.001)	−
Age group (in years)	< 60	118 (25.3)	−	−
	≥ 60	348 (74.7)	2.14 (1.56–2.92; *p* < 0.001)	1.87 (1.35–2.60; *p* < 0.001)
Race	White	208 (44.6)	−	−
	Black	89 (19.1)	1.32 (0.97–1.82; *p* = 0.081)	−
	Non-white/black	169 (36.3)	1.20 (0.92–1.56; *p* = 0.187)	−
Race	White	208 (44.6)	−	−
	Non-white	258 (55.4)	1.24 (0.98–1.57; *p* = 0.078)	−
2018 FIGO	I	209 (44.8)	−	−
	II	58 (12.4)	2.18 (1.47–3.25; *p* < 0.001)	2.65 (1.75–4.01; *p* < 0.001)
	III	139 (29.8)	3.31 (2.46–4.45; *p* < 0.001)	4.67 (3.17–6.86; *p* < 0.001)
	IV	60 (12.9)	7.01 (4.91–10.01; *p* < 0.001)	9.75 (6.22–15.29; *p* < 0.001)
ECOG-PS	0	116 (25.3)	−	−
	1	297 (64.8)	2.80 (1.99–3.95; *p* < 0.001)	1.90 (1.33–2.72; *p* < 0.001)
	2	33 (7.2)	4.13 (2.53–6.75; *p* < 0.001)	2.29 (1.36–3.84; *p* = 0.002)
	3	12 (2.6)	6.62 (3.39–12.95; *p* < 0.001)	2.83 (1.36–5.87; *p* = 0.005)
Myometrium invasion	No invasion	24 (5.2)	−	−
	Less than 50%	214 (46.0)	1.18 (0.63–2.20; *p* = 0.600)	−
	More than 50%	227 (48.8)	2.33 (1.26–4.30; *p* = 0.007)	−
Myoinvasion	No or less than 50%	238 (51.2)	−	−
	More than 50%	227 (48.8)	2.01 (1.58–2.55; *p* < 0.001)	1.53 (1.17–2.00; *p* = 0.002)
Histological subtype	G3EA	145 (31.1)	−	−
	USC	118 (25.3)	1.95 (1.41–2.68; *p* < 0.001)	1.53 (1.09–2.17; *p* = 0.015)
	CCC	44 (9.4)	1.43 (0.92–2.23; *p* = 0.110)	1.10 (0.69–1.74; *p* = 0.696)
	CCS	63 (13.5)	2.81 (1.94–4.08; *p* < 0.001)	2.19 (1.47–3.27; *p* < 0.001)
	Mixed	93 (20.0)	1.13 (0.78–1.64; *p* = 0.533)	0.99 (0.66–1.47; *p* = 0.946)
	NOS	3 (0.6)	0.00 (0.00–Inf; *p* = 0.990)	0.00 (0.00-Inf; *p* = 0.992)
Treatment	No treatment	104 (22.4)	−	−
	Chemotherapy with or without EBR/BT	235 (50.5)	0.82 (0.62–1.09; *p* = 0.178)	0.31 (0.22–0.46; *p* < 0.001)
	EBR and/or BT	126 (27.1)	0.44 (0.31–0.63; *p* < 0.001)	0.48 (0.32–0.70; *p* < 0.001)

Abbreviations: 95%CI, 95% confidence interval; BT, brachytherapy; CCC, clear-cell carcinoma; CCS; carcinosarcoma; EBR, external beam radiotherapy; ECOG-PS, Eastern Cooperative Oncology Group's Performance Status scale; FIGO, Fédération Internationale de Gynécologie et d'Obstétrique (International Federation of Gynecology and Obstetrics, in French) staging system; G3EA, grade-3 endometrioid adenocarcinoma; HR, hazard ratio; Mixed, mixed epithelial tumors; NOS, not otherwise specified; SD, standard deviation; USC, uterine serous carcinoma.

## Discussion

The diagnosis of EC is usually established in patients with less aggressive histologies and early-stage disease (type I tumors), which leads to a high chance of cure with surgery alone. The incidence is increasing worldwide due to the increasing rate of obesity and the aging of the population. 2 In Brazil, the incidence is higher in more developed areas, such as the Southeastern region, and is also expected to increase. ^1^ Type-II EC, such as the histologies analyzed in this cohort, has long been known for its poor prognosis, and multimodality treatment is usually recommended by major international guidelines. 10 11

Important insights can be drawn based on the present retrospective and descriptive analysis. First, we were able to reinforce the international data regarding the poor prognosis of high-risk histologies and the prognostic impact of higher stages. 5 Although we did not collect data about low-grade endometroid tumors (FIGO stages I and II), we could conclude the poorer outcome of our cohort compared with the former based on the international literature. In the present analysis, serous carcinoma and CCS retained a poor prognosis compared with high-grade endometrioid carcinoma. This finding contrasts with those of some studies, but it is also in line with those of many others. 5 6 7 8 In an analysis done by the Canadian High-Risk Endometrial Cancer (CHREC) Consortium with 1,260 women with high-risk histologies (grade-3 endometrioid adenocarcinoma [G3EA], CCC, CS, CCS), 5 the distribution of FIGO stages was similar to that of our cohort (49.3% versus 44.8% for stage I; 10.6% versus 12.4% for stage II; 27.4% versus 29.8% for stage III; and 12.7% versus 12.9% for stage IV). Regarding adjuvant treatment, 74.5% of the patients in the Canadian study and 77% in the present analysis underwent one treatment modality, with similar use of chemotherapy (54.8% versus 50% respectively). In both studies, CCS had the worse prognosis, with shorter OS and RFS among the high-risk histologies. 5 In our cohort, we observed more recurrences than in the Canadian cohort (43.9% versus 31.5% respectively), but the distribution was similar, with most recurrences in distant sites. A second point must be made regarding surgical aspects. International prospective randomized clinical trials 12 13 14 have highlighted the safety and lower morbidity of the minimally-invasive surgery (MIS) for EC, and have also showed safety in subgroup analyses for high-grade histologies. Although these trials included mainly low-grade endometrioid tumors (stage I), MIS was seldom used in our cohort despite the rate of 45% of patients in stage I. There is only one robotic platform at INCA, which was installed in 2012, and only one platform for laparoscopic surgery at the Gynecologic Oncology Unit. The low rates of MIS can also be explained by the fact that not every surgeon is certified to perform robotic surgery and by the frequent lack of surgical supplies. In addition, although lymph node assessment is recommended for any EC with myoinvasion, with sentinel lymph node being the standard of care for the uterine confined disease, about a third of the patients did not undergo any lymph node assessment. Unfortunately, we could not collect data regarding the use of sentinel lymph node. Third, the importance of treating patients according to guidelines is well described in the literature. 10 11 15 16 The cohort of the present study was composed of high-risk patients, to whom the adjuvant treatment is almost always recommended, especially chemotherapy, and 22% percent of the patients did not undergo any kind of adjuvant treatment. Undergoing any type of adjuvant treatment was associated with better survival than observation in the present analysis. Intriguingly, most recurrences were in distant or abdominal sites, which puts into question the role of adjuvant external beam radiation for these patients.

The present analysis has several limitations, mainly based on its inherent retrospective nature, with many confounding factors that cannot be addressed, for it involved a single-center population and issues regarding the accuracy of the data collection. There were also limited data on comorbidities, which could have impacted the survival, and we were not able to classify the patients based on the new molecular classification. The strength of the study is that it involved a large number of patients with high-risk histology.

## Conclusion

We retrospectively reviewed a cohort of patients with high-risk histologies treated at INCA between 2010 and 2016. We reinforced the international data showing the poor outcomes of these tumors, especially for serous carcinoma and CCS and tumors with advanced stages, with shorter survival and high recurrence rates in distant sites, independently of the FIGO stage. Also, as recommended by the guidelines, adjuvant therapy was associated with better survival.
